# Autophagy-dependent danger signaling and adaptive immunity to poorly immunogenic tumors

**DOI:** 10.18632/oncotarget.13892

**Published:** 2016-12-10

**Authors:** Guido Kroemer, Lorenzo Galluzzi

**Affiliations:** ^1^ Equipe 11 Labellisée par la Ligue Contre le Cancer, Centre de Recherche des Cordeliers, Paris, France; ^2^ INSERM, U1138, Paris, France; ^3^ Université Paris Descartes/Paris V, Sorbonne Paris Cité, Paris, France; ^4^ Université Pierre et Marie Curie/Paris VI, Paris, France; ^5^ Metabolomics and Cell Biology Platforms, Gustave Roussy Comprehensive Cancer Institute, Villejuif, France; ^6^ Pôle de Biologie, Hopitâl Européen George Pompidou, AP-HP, Paris, France; ^7^ Department of Women’s and Children’s Health, Karolinska University Hospital, Stockholm, Sweden; ^8^ Gustave Roussy Comprehensive Cancer Institute, Villejuif, France; ^9^ Department of Radiation Oncology, Weill Cornell Medical College, New York, NY, USA

**Keywords:** ATP, chloroquine, damage-associated molecular patterns, doxorubicin, immunogenic cell death, radiation therapy

## Abstract

Recent data suggest that autophagy does not influence spontaneous and therapy-elicited tumor infiltration by immune cells in murine models of melanoma and breast carcinoma. These findings, which have been obtained in the absence of a therapeutically relevant anticancer immune response, indicate that the intrinsically low immunogenicity of some tumors cannot be compensated for by increased danger signaling.

Macroautophagy (hereafter referred to as autophagy) is a highly conserved catabolic pathway though which eukaryotes preserve homeostasis at both the cellular (cell-intrinsic) and organismal (cell-extrinsic) level [1, 2]. Thus, autophagy continuously operates at low rates to remove cytoplasmic entities that may accumulate (and hence pose a threat) as a consequence of normal metabolism, such as redox-active protein aggregates and permeabilized mitochondria [3]. Moreover, the flux of substrates through the autophagic machinery is highly responsive to perturbations of intracellular and extracellular homeostasis as diverse as nutritional, metabolic, hormonal, physical and chemical cues [4-6]. This means that most - if not all - eukaryotic cells can adapt the rate of autophagic degradation to external stimuli, and this is a key component of adaptive stress responses [7]. In line with this notion, inhibiting autophagy with pharmacologic agents or genetic maneuvers generally precipitates (rather than retards) the death of cells experiencing potentially lethal microenvironmental perturbations [8, 9]. Finally, autophagy contributes to the preservation of organismal homeostasis and supports healthy aging as it impacts on multiple extracellular processes with local (short-range) or systemic (long-range) outcomes [10, 11]. As a standalone example, proficient autophagic responses in muscles and in the liver are required for the beneficial effects of endurance exercise on systemic glucose metabolism [12].

Unfortunately, the pronounced capacity of autophagy to support cellular homeostasis in the course of adaptive stress responses does not benefit normal, healthy cells only [3, 13]. Thus, autophagy not only promotes natural tumor progression as it favors the survival of malignant cells experiencing adverse microenvironmental conditions (e.g., hypoxia, reduced nutrient and growth factor availability) [14], but also promotes chemo-and radioresistance (at the cell-intrinsic level) [15, 16]. Accordingly, chemical agents as well as genetic interventions targeting core components of the autophagic machinery have been shown to increase the sensitivity of cultured cancer cells to a wide panel of chemotherapeutic agents and radiotherapeutic regimens [15]. Similar findings have been obtained when the response of autophagy-competent *versus* autophagy-deficient cancer cells to treatment was evaluated in immunodeficient animals [15]. Throughout the past decade, all these observations generated remarkable enthusiasm on the possibility that inhibiting autophagy would mediate anticancer effects *per se*, or it would boost the efficacy of conventional chemo- and radiotherapeutic regimens [2, 17]. The results of multiple clinical trials testing these therapeutic paradigms in cancer patients, however, have been largely disappointing [18-25].

Although several factors may underlie such a fiasco, we and others are persuaded that it relates for the most part to: (1) the too-often-disregarded cell-extrinsic effects of autophagic responses within malignant cells, and (2) the cell-intrinsic effects of autophagy in non-malignant components of the tumor environment (including immune effector cells) [15]. Proficient autophagic responses in neoplastic cells succumbing to some chemotherapeutics are indeed fundamental for optimal danger signaling, which involves the spatiotemporally ordered emission of endogenous immunostimulatory molecules commonly referred to as “damage-associated molecular patterns” (DAMPs) [26-29]. In particular, autophagy-deficient malignant cells fail to secrete ATP and to release high-mobility group box 1 (HMGB1) as they die, underlying their inability to trigger tumor-targeting immune responses in the absence of exogenous adjuvants [26, 27, 30, 31]. Moreover, the activation of autophagy in dying cancer cells is required for dendritic cells to optimally process tumor-associated antigens and cross-present them to CD8+ cytotoxic T lymphocytes (CTLs) on MHC Class I molecules [32-34]. Thus, autophagy-deficient malignant cells growing in syngeneic immunocompetent hosts are generally less (rather than more) sensitive to anticancer agents that actively promote a therapeutically relevant immune response, including doxorubicin and oxaliplatin [35, 36], as well as to some forms of radiotherapy [37- 39], then their autophagy-competent counterparts [26, 27, 40, 41]. Finally, autophagy is required for the survival, proliferation and activity of multiple cell populations involved in adaptive anticancer immunity, notably CD8+ CTLs [42-46]. Taken together, these observations suggest that not only the systemic but also the local inhibition of autophagy, may limit the efficacy of antineoplastic agents that stimulate anticancer immune responses [47], at least in immunocompetent hosts (such as the vast majority of cancer patients). As a corollary of this hypothesis, it has been proposed that activating (rather than inhibiting) autophagy may boost the efficacy of cancer therapy [15]. Indeed, various nutritional interventions that potently trigger autophagy in multiple organs (including short-term fasting, fasting-mimicking diets, and so-called caloric restriction mimetics, CRMs) turned out to improve disease outcome in a variety of rodent tumor models established in syngeneic immunocompetent hosts [40, 48-51]. Moreover, biomarkers of proficient autophagic responses in malignant cells have been associated with improved disease outcome in cohorts of breast carcinoma and multiple myeloma patients [52-54]

Irrespectively (and in spite of the considerable success achieved by multiple forms of immunotherapy throughout the past decades), many investigators and clinicians tend to perceive cancer as a cell-autonomous disorder, and hence favor the interpretation that autophagy should be inhibited in the context of cancer therapy [2]. To obtain additional insights into this issue, Starobinets and collaborators recently investigated the impact of autophagy inhibition on quantitative and qualitative aspects of the immunological tumor infiltrate in two mouse tumors models, notably B16 melanoma cells (syngeneic to C57BL/6 mice) and 4T1 breast carcinoma cells (syngeneic to BALB/c mice) implanted subcutaneously or orthotopically [55].

Initially, B16 and 4T1 cell variants with stable (but partial) autophagic defects imposed by the short hairpin RNA (shRNA)-mediated depletion of autophagy related 7 (Atg7) or autophagy related 12 (Atg12) were established. The stable downregulation of Atg7 or Atg12 did not affect tumor growth in immunocompetent syngeneic mice [55], contrasting with previous literature on the topic [15]. Next, spontaneous tumor infiltration by multiple populations of immune cells (CD45+ cells, CD3+ T cells, CD3+CD4+ T cells, and CD3+CD8+ CTLs) [56] was determined in established (2-3 weeks after implantation) autophagy-competent *versus* -incompetent tumors, and no differences were found [55]. Similarly, the spontaneous CD3+CD4+ and CD3+CD8+ T-cell infiltrates isolated from autophagy-proficient *versus* -deficient B16 and 4T1 tumors growing in immunocompetent syngeneic mice did not differ with respect to an panel of activation/exhaustion markers encompassing CD44, interferon gamma (IFNG), tumor necrosis factor (TNF), granzyme B (GRZB, measured on CD8+ CTLs only) and programmed cell death 1 (PDCD1, best known as PD-1) [55, 57, 58].

To extend their observations to another relevant model, Starobinets and colleagues generated autophagy-competent (transfected with an irrelevant shRNA) and -incompetent (transfected with Atg7- and Atg12- targeting shRNAs) B78 melanoma cells stably expressing ovalbumin (OVA) as a model antigen [55, 59]. OVA-expressing autophagy-proficient and -deficient B68 tumors developed with similar kinetics in immunocompetent C57BL/mice, and they attracted comparable amounts of adoptively transferred OVA-specific CD3+CD4+ and CD3+CD8+ OT-1 cells [55, 60]. Moreover, CD3+CD4+ and CD3+CD8+ OT-1 cells infiltrating autophagy-competent *versus* -incompetent B68 tumors did not differ with respect to CD44, IFNG, TNF and GRZB expression [55].

Next, the release of ATP and HMGB1 by autophagy-proficient *versus* -deficient B16 cells exposed to doxorubicin *in vitro* was monitored, confirming that doxorubicin can promote the release of these DAMPs by B16 cells in an autophagy-dependent manner. However, Starobinets and collaborators could not vaccinate C57BL/6 mice with B16 cells succumbing to doxorubicin, irrespective of autophagic proficiency [55, 61, 62]. This in line with a previous report from our group, demonstrating that B16 cells are poorly immunogenic (repeated administrations of dying B16 cells was required to achieve protective immunity, to some extent) [63]. Of note, intravenous doxorubicin failed to limit the growth of autophagy-competent (and -incompetent) B16 tumors established in C57BL/6 mice. Moreover, the differential sensitivity of autophagy-proficient *versus* -deficient B16 cells to acute cell death induced doxorubicin *in vitro* was marginal at best (same sensitivity at 8 different concentrations of the drug in the 1-10 μM and 20-100 μM ranges, some extent of increased sensitivity for Atg7- depleted cells at approximately 1, 10 and 50 μM; based on a single representative experiment) [55]. In the absence of any therapeutic effect, doxorubicin failed indeed to promote the infiltration of B16 tumors by CD3+CD4+ and CD3+CD8+ T cells, irrespective of autophagic-proficiency, although it did promote some activation in the pre-existing tumor infiltrate (as per intratumoral levels of CD3+CD4+CD44+ and CD3+CD8+CD44+ cells [55].

Finally, Starobinets and colleagues monitored the impact of autophagy inhibition with antimalarial drugs like chloroquine and quinacrine (which are highly non-specific as they block lysosomal degradation) [64-68] on the growth of B16 and 4T1 cells in immunocompetent hosts [55]. Neither chloroquine nor quinacrine had an effect on tumor progression *in vivo* (although they did inhibit autophagy in malignant cells). Moreover, these antimalarial drugs failed to affect spontaneous tumor infiltration by CD3+CD4+ and CD3+CD8+ T cells and the activation/exhaustion status of these cells [55 Apetoh, 2015, 26137416]. It is tempting to speculate that such a complete absence of response reflects (at least in part) the detrimental effects of autophagy inhibition on various populations of the immune system (including myeloid antigen-presenting cells, a compartment for which autophagy is particularly important from a functional perspective) [69-74].

In conclusion, the findings by Starobinets and colleagues suggest that autophagy-dependent DAMP signaling cannot compensate for the intrinsically low immunogenicity of some tumors (and the consequent absence of a therapeutically relevant anticancer immune response). An abundant literature demonstrates that autophagy plays a critical role in the capacity of multiple chemotherapeutic and some forms of radiation therapy to elicit anticancer immune responses that beneficially influence disease outcome (in mice and in cancer patients). Thus, in tumor models in which chemotherapy (or radiation therapy) mediates antineoplastic effects that depend on the immune system, the autophagic proficiency of malignant cells appears to support the elicitation of local, therapeutically relevant anticancer immune responses [75-79]. Moreover, in such models, nutritional interventions that potently induce autophagy at the whole-body level can boost the efficacy of chemotherapy or radiation therapy via immunological mechanisms. These experimental settings include (but are not limited to) (1) mouse MCA205 fibrosarcomas, TC1 lung carcinomas, 4T1 breast carcinomas, and CT26 colorectal cancers established in immunocompetent syngeneic mice; (2) carcinogen-driven breast carcinomas in C57BL/6 mice; and (3) KRASG12D-induced lung carcinoma in in C57BL/6 mice [40, 50]. In sharp contrast, as demonstrated by Starobinets and colleagues [55], when chemotherapy fails to elicit immunogenic cell death, the autophagic proficiency of malignant cells does not influence anticancer immunosurveillance (which is therapeutically irrelevant *a priori*).
